# We Matter: Pilot Study on the Impact of Asian, Pacific Islander, and Desi-American (APIDA) Stories of Mental Illness to Address Stigma

**DOI:** 10.1007/s10597-025-01546-w

**Published:** 2025-11-03

**Authors:** Jennifer T. Tran, Cassidy Bolton, Vivian Ko, Claudia Matteo, Kristin Kosyluk

**Affiliations:** 1 University of Pennsylvania, Philadelphia, United States; 2 University of South Florida, Tampa, United States; 3 Florida State University, Tallahassee, United States; 4 This Is My Brave, Leesburg, United States

**Keywords:** Stigma reduction, Contact-based intervention, Storytelling, Asian/asian american

## Abstract

Asian Americans (AA) have experienced increased rates of serious mental illness over the past decade. Past research has identified perceived and personal mental illness stigma as significant barriers to seeking treatment for mental health concerns, particularly in the Asian American community. One way to address stigma has been through narratives told from the perspective of community members of a stigmatized identity. Therefore, this study examines the impact of This Is My Brave: Stories from the Asian, Pacific Islander, and Desi American Community (TIMB: SAC; a narrative-based stigma reduction intervention) on audience members. Participants (*N* = 89; mean age = 27.62[SD = 9.82]) had a significant increase in intentions to seek care and a significant decrease in personal mental illness stigma, perceived mental illness stigma, and anti-Asian American stereotypes from pre-intervention to post-intervention. We did not find any significant differences between AA and non-AA individuals on any of the dependent measures (personal stigma, perceived stigma, anti-racism, anti-Asian attitudes, and intentions to seek care). This study has implications for TIMB: SAC as a stigma reduction intervention.

## Introduction

Nearly 60 million U.S. adults (23% of the population in 2022) experience mental illness every year, and over 49,000 die by suicide, 90% with a diagnosable psychiatric disorder (U.S. Centers for Disease Control and Prevention [CDC], 2025; Substance Abuse and Mental Health Services Administration [SAMHSA], 2019a). Serious mental illness (SMI) rose from 2.9% to 5.6% in Asian Americans (AA) and Pacific Islanders ages 18–25 between 2008 and 2018 (SAMHSA, 2019b). About 3.5% of Pacific Islander adults experienced a serious mental illness within the past year (SAMHSA, 2019b), and 20% of Desi Americans experience a mood or anxiety disorder (Masood et al., 2009). Major depressive episodes increased in all AA community members (which encompasses Asian, Pacific Islander, and Desi Americans; APIDA) across all age groups from 2015 to 2018 (SAMHSA, 2019b). Suicidal thoughts, plans, and attempts also rose among AA young adults (SAMHSA, 2019b). It is important to note that the AA community represents an ethnically, linguistically, and culturally diverse group; however, there is limited research on the differences in mental health (Bhakta, 2022).

The Asian American community encompasses a largely heterogeneous ethnic and cultural population. For our understanding, we define and highlight the following terms for subgroups: (1) Asian Americans include people originating from China, the Philippines, Vietnam, Korea, and other countries in East and Southeast Asia, (2) Pacific Islanders include people from Hawaii, Guam, and Samoa, and (3) Desi Americans include people originating from South Asia, such as India, Bangladesh, and Pakistan (The Asian American Education Project, n.d.). We differentiate and call out Desi Americans and Pacific Islanders as specific subgroups of the Asian Americans umbrella due to the historical underrepresentation of these groups (The Asian American Education Project, n.d.).

Despite the research on the increase in incidence and prevalence of mental health conditions in the AA community, the AA community has the lowest rates of mental health service utilization among all racial/ethnic minority groups (Lee & Howard, 2022; Lu et al., 2024). At the same time, AA members are the least likely racial group to act on their mental health and are more likely to reach out to friends and family (Spencer et al., 2010). However, not all AA members have a robust support system and can have difficulty expressing their challenges due to guilt, shame, or even the inability to speak the same language (Sangalang & Gee, 2012; Spencer & Chen, 2004). Among multiple explanations, stigma has been identified as one of the most powerful barriers to mental healthcare (Spencer & Chen, 2004).

Some form of stigma experienced by AA members is perceived public stigma (Corrigan et al., 2014a, b, c; e.g., one’s perception of how stigmatizing others in their community are towards mental illness which can lead to the fear of being treated differently, being devalued or discriminated against by others due to having a mental illness) and internalized stigma (Corrigan et al., 2009; e.g., feelings of shame, guilt and diminished self-esteem), and it deters people from seeking mental health care when they need it (Watson et al., 2007). Unfortunately, research suggests that mental illness stigma is amplified within the AA community (APA, 2012). Within the AA community and culture, mental illness has been described as a Western concept, is not talked about within AA culture, and even in some cases is culturally erased (i.e., seen as something that doesn’t exist; Augsberger et al., 2015). Culturally relevant strategies are desperately needed to reduce stigma and improve knowledge and attitudes about seeking treatment that can impact these disparities in service utilization in the APIDA community.

### Anti-Asian Racism

The United States embodies a racial hierarchy that enforces privilege for Whiteness and discriminates against non-Whites, such as the APIDA communities (Chang, 2015). Asian Americans continually experience discrimination because of their racial minority status and are particularly impacted by two prominent stereotypes: the model minority myth and the perpetual foreigner (Hwang, 2021; Kim et al., 2023; Yi et al., 2023). On the surface, the model minority myth suggests high academic and economic achievements among the group; however, it oversimplifies the diverse experiences within the AA community. This myth can lead to the erasure of struggles and challenges faced by individuals within the community and can place immense pressure on this population to conform to perceived standards (Hwang, 2021; Yi et al., 2023). The perpetual foreigner stereotype occurs when individuals in the AA community are asked where they are from, and the response of a city or state in the United States does not satisfy the question. This prompts the follow-up question of “Where are you really from?”, reinforcing the idea that Asian Americans are not considered Americans (Hwang, 2021; Pew Research Center, 2023). These two stereotypes have increased the number of reports of Asian American racial discrimination and incidents as seen during and following the COVID-19 pandemic.

### Internalized Racism

Some community members internalize the model minority myth, which may seem to have positive connotations; however, it is rooted in and associated with the myth of white supremacy, including the denial of racial barriers and inequalities. Prior research has shown that there are links between internalized model minority myth and negative psychological outcomes of distress and lower mental health help-seeking (Atkin et al., 2018). To cope with their own experiences of racism and discrimination while growing up in predominantly White communities, some AA youth have participated in self-mockery, defensive othering, and disassociating themselves from others within their ethnic group (Trieu & Lee, 2018). Although these internalized racial oppression practices were employed to help these youth fit in as racial minorities in their community, they also perpetuated the negative stereotypes. Often, these behaviors were used to distance individuals from the negative stereotypes of the ethnic group in the hopes of being accepted into the dominant group (Pyke, 2010). Additionally, internalized racism can create divisions within ethnic groups and lead to negative self-perception (Hwang, 2021).

### Stigma-Reduction Strategies

Evidence-based stigma-reduction approaches include protest, education, and contact (Corrigan et al., 2012), with research showing contact-based approaches (e.g., personal interaction with individuals living with a mental illness) having small to medium effects in changing mental illness stigma (Corrigan et al., 2012; Corrigan & Kosyluk, 2014; McCullock & Scrivano, 2023; Morgan et al., 2018). According to Allport’s Intergroup Contact Theory (1954), direct contact between members of different social or cultural groups (out-group, members of the marginalized group, and in-group, members of the dominant society) can promote mutual understanding and reduce prejudice. Therefore, contact-based interactions involve individuals with mental illnesses sharing their stories of lived experience (including their challenges with mental illness and the associated symptoms, experiences with stigma, and their recovery journey, and messages of hope) with members of the in-group (individuals who may hold prejudicial views towards individuals with mental illness). Furthermore, contact-based strategies to reduce stigma are more effective when in-group members identify with out-group members. Research suggests that people are more responsive to contact-based stigma reduction strategies if the message is delivered by someone they can relate to (Corrigan, 2011; Davidson et al., 2018). Differences in experiences of stigma, therefore, suggest culturally adapted contact stigma reduction strategies to address AA mental health stigma are crucial. Well-developed interventions have the potential to impact attitudes towards mental illness and mental health treatment (Simmons et al., 2017).

### This is my Brave

Run by people with lived mental illness experience, This Is My Brave (TIMB) is a nonprofit organization whose mission is to empower individuals to put their names and faces on their true stories of recovery from mental illness and addiction. TIMB produces shows nationwide and abroad, to address stigma by featuring local community members in each location who share their personal stories of overcoming mental illness and addiction to lead full lives, and they do this through creative expression (e.g., poetry, song, dance, storytelling, comedy). The TIMB shows are funded by the community members and assisted by the TIMB organization through fundraising efforts. (More information on TIMB can be found at thisismybrave.com)

Research shows TIMB effectively reduces public stigma and discrimination, improving beliefs about recovery from mental illness and improving attitudes toward treatment-seeking (Kosyluk et al., 2018, 2021). In a special edition show, TIMB, community partners, and key researchers developed TIMB: Stories from the Black Community (TIMB: SBC). TIMB: SBC was found to decrease perceived stigma, personal stigma, and discrimination and increase antiracism, overall attitudes towards mental health treatment, and beneficial attitudes towards mental health treatment in audience members (Conner et al., 2023).

### This is my Brave: Stories from the Asian, Pacific Islander, and Desi-American (APIDA) Community (TIMB:SAC)

There is a lack of evidence on the use of stigma reduction programs to aid in reducing mental illness-related stigma in Asian American communities. Therefore, based on previous work demonstrating the effectiveness of contact-based stigma change approaches and the increase in racial discrimination and violence against AA members in the United States, a special TIMB show was developed entitled: *“This Is My Brave: Stories from the Asian*,* Pacific Islander*,* and Desi American (APIDA) Community (TIMB: SAC).”*

#### Current Study

This study evaluates a culturally meaningful intervention to address mental health stigma and anti-Asian attitudes impacting the Asian American community. There has not been a targeted effort to address issues of shame, stigma, cultural values, and recent experiences of racism in the Asian American community via this contact-based stigma change program. We hypothesize that *all viewers* of the virtual TIMB: SAC show will:

1. Experience significantly reduced mental illness stigma.
2. Exhibit a significant improvement in intentions to seek care.
3. Experience a significant reduction in negative racial stereotypes and attitudes toward the Asian American community.

Furthermore, we expect to see differences in the impact of the culturally adapted show, TIMB: SAC, on *AA viewers compared to non-AA viewers*. Based on prior literature, participants who feel connected to the storytellers have greater reductions in stigma (Conner et al., 2023; Corrigan & Kosyluk, 2014). As the TIMB: SAC show specifically highlights Asian, Pacific Islander, and Desi American stories, we will purposively examine viewers who also identify within these subgroups to avoid underrepresentation. Therefore, Asian American viewers (including Pacific Islanders and Desi Americans) may feel more connected to storytellers from the TIMB: SAC. We hypothesize that Asian American viewers will:

1. Exhibit a more significant reduction in mental illness stigma.
2. Exhibit a greater improvement in intentions to seek care.
3. Experience a greater reduction in negative racial stereotypes and attitudes toward Asian American viewers compared to non-Asian American viewers.

## Methods

The study examines the impact of TIMB: SAC on mental illness stigma, intentions to seek care, and attitudes towards Asian Americans among audience members (AA viewers and non-AA viewers). Although the Asian American population encompasses considerable heterogeneity across ethnic, cultural, and socioeconomic dimensions, the constraints imposed by the limited sample size and exploratory design of this pilot study necessitated the aggregation of all Asian American participants into a single analytical category.

### TIMB: SAC

The development of TIMB: SAC is not included in the current study; however, information on the show is provided here for context. TIMB: SAC show was inspired by a conversation between the TIMB Co-Founder and an Asian American TIMB Board Chair. A national call was put out via a flyer distributed by the production team and staff through various social media platforms to audition APIDA individuals living with a mental illness or addiction to share their stories on the TIMB platform—a dozen individuals auditioned by submitting a video of themselves sharing their stories through creative means.

The selection of cast members was based on the representation of a diverse mix of cultures and identities across age, ethnicity, gender, sexual orientation, mental health conditions, neurodiversity, and veteran status to convey that the APIDA community is not a monolith. Diverse representation and intersectional identities also help expand the reach across audience members, as the experiences and identities of each cast member resonate with a subset of the audience who can relate. Eight cast members were chosen to be a part of the special show. Cast members are diverse in their racial and ethnic identities of Asian American, Pacific Islander, and Desi American. At least half of the cast members were also mixed-race, identifying as half-Asian and half-White. The production team virtually worked with the storytellers over six rehearsals (four rehearsals, one check-in, and one tech rehearsal) to finalize performances that included songs, spoken word, and monologues. Storytellers recorded their final versions and then edited stories for a virtual show. The opening, closing, and cast panel discussion portions were live virtually. The TIMB: SAC show was a total of 60 min. (More information about the show can be found at https://thisismybrave.org/apida/).

### Sample and Study Procedures

Participants were purposively sampled for a diverse range of Asian American, Desi American, and Pacific Islanders, as we hypothesized that TIMB: SAC would have a greater impact in reducing stigma for participants who identified with our APIDA storytellers. We specifically attempted to target these three subgroups due to the historical underrepresentation of Desi Americans and Pacific Islanders in studies. Study participants were recruited in four ways: (1) people registered to watch TIMB: Stories from the APIDA Community Show live virtually (*n* = 4), which was marketed via social media and through This Is My Brave over the three months leading up to the event, (2) students at the university where the study took place (*n* = 36) were contacted through university listservs, and flyers sent out to classes and registered to watch the virtual show, (3) through APIDA community organizations with high numbers of APIDA minority participants (*n* = 3) were contacted via in-person and email conversations, and (4) online through Prolific (*n* = 46) pre-screening for individuals that identify as APIDA to ensure adequate diversity in our sample as our interest was to compare measures between groups that identify as APIDA and not. Participants who registered for the live show and those that were recruited from community partners received $15 Amazon gift card for completing the study. Participants recruited through Prolific were given compensation for the completion of the study (pre-survey, watching the video, and post-survey) of $14.25. Student participants were offered extra credit in their courses for completing the study, while students who did not wish or were ineligible to participate in the study were offered an alternative assignment for the same number of extra credits. All participants completed a pre-survey (either within 24 h before or right before the show) and then watched the TIMB: SAC show either synchronously or asynchronously virtually. The TIMB: SAC show lasted about 60 min. Directly after watching the show, participants also completed a post-show survey.

This study was submitted to our university’s institutional review board and was deemed exempt due to its minimal risk. However, prior to completing the baseline survey, participants were still provided with informed consent detailing the study, benefits and risks, and information privacy. Participants were asked to consent to taking the survey before being able to continue.

### Measures

Measures included demographic information, mental illness stigma measures (e.g., the Attribution Questionnaire for personal stigma (AQ-9; Corrigan et al., 2014a, b, c), the Perceived Devaluation-Discrimination Scale (PDDS; Link et al., 2001) for perceived stigma), Anti-Asian American Stereotypes with the Scale of Anti-Asian American Stereotypes (SAAAS; Lin et al., 2005), and intentions to seek care with the Care Seeking Questionnaire (CSQ; Corrigan et al., 2013).

#### Demographic Information

Participants were asked demographic questions in the pre-survey, which included questions on self-reported race, ethnicity, gender, sexual orientation, education, employment status, marital status, and mental health diagnoses and treatment. Differentiation of participants as Asian American and non-Asian American was based on a question asking, “Which group do you most closely identify with?” Participants who selected either Asian/Asian American, Pacific Islander, or Desi American were coded as AA, and all others were coded as non-AA.

#### Personal Stigma

Personal stigma is an individual’s attitudes, stereotypes, prejudices, and behaviors toward individuals living with mental health conditions. Personal stigma was measured using the Attribution Questionnaire (AQ-9), which has good internal consistency (α = 0.73), test-retest reliability (*r* =.73), and construct validity (Corrigan et al., 2014a, b, c). The AQ-9 includes nine questions following the vignette about a man, Harry, living with schizophrenia, which is answered on a nine-point Likert scale. An example item is, “How dangerous would you feel Harry is?” Higher scores on the AQ-9 indicate higher levels of personal stigma.

#### Perceived Stigma

Perceived stigma refers to one’s perceptions of how stigmatizing their community is toward a person with a mental health condition. Perceived stigma was measured using the Perceived Devaluation-Discrimination Scale (PDDS; Link et al., 2001). The scale has demonstrated good reliability, with alphas ranging from 0.86 to 0.88 (Link et al., 2001) and validity. Studies show a relationship between demoralization and PDDS scores for individuals with a psychiatric label (Link et al., 1989). The PDDS is a 12-item instrument asking participants to indicate how they agree with statements on their community perceptions towards individuals with mental health conditions. An example item reads, “Most people feel that entering a psychiatric hospital is a sign of a personal failure.” Items are responded to on a six-point Likert scale, with higher scores on the PDDS representing greater perceived public stigma.

#### Anti-Asian American Stereotypes

Anti-Asian American stereotypes were measured using the Scale of Anti-Asian American Stereotypes (SAAAS), which is a measure that has been found to have good internal consistency (α = 0.93) and contains two subscales: (Un)Sociability (α = 0.90) and Competence (α = 0.86; Lin et al., 2005). The SAAAS includes 25 items answered on a 5-point Likert scale (5 = strongly agree). The SAAAS is based on the Stereotype Content Model (SCM), which posits that outgroups fall within a range between competence and sociability. Competency reflects capability and high status, while sociability encompasses warmth and likeability. Outgroups may be perceived as one or the other, but not both (Lin et al., 2005). An example item is, “Asian Americans seem to be striving to become number one.”

#### Intentions to Seek Care

Intentions to Seek Care surrounding mental health treatment will be measured using the Care Seeking Questionnaire (CSQ). The CSQ is a 6-item measure responded to on a 9-point Likert scale (9 = strongly agree). An example item is, “I would speak to a psychiatrist if I were significantly anxious or depressed.” The CSQ has demonstrated satisfactory reliability and validity (Corrigan et al., 2013).

### Intervention Attention Check

After watching the show virtually, participants were asked attention-check questions that had to be answered correctly to receive the post-show survey and compensation. They were asked two content questions about different storytellers from the TIMB: SAC that could only be answered if the participant was watching the show. For example, one question asked what the name of a storyteller’s act was called.

### Data Analysis

All analyses were performed in SPSS Version 28 (IBM Corp., 2019). Descriptive statistics were used to describe the sample, and paired samples t-tests were used to assess changes in variables from pre- and post-intervention. A priori statistical power analysis was performed for sample size estimation using G*Power3.1 (Faul et al., 2009) for a paired-samples t-test, with a moderate effect size (d = 0.50) and an alpha of 0.05. Results showed that a total sample size of 45 participants was required to achieve a power of 0.95.

ANOVAs and chi-square tests were used to test for significant differences between groups regarding demographics (AA v non-AA). However, we chose to combine these groups and not control for demographic differences in the following analysis because we looked for interactions on outcomes by demographic variables. Also, as this was a pilot and not an RCT, our goal was to attain preliminary data on the impact of this intervention and not to attain equally balanced groups. Further, all outcomes were in the expected direction for each sample individually. ANCOVAs were used to assess change over time (pre-test/post-test) on all outcome variables (personal stigma, perceived stigma, anti-racism, anti-Asian stereotypes, and intentions to seek care) for AA participants vs. non-AA participants. Based on theoretical understanding of differences in stigma experiences, we controlled for age, gender, sexual orientation, education level achieved, relationship status, previous diagnosis, past treatment, and current treatment.

## Results

### Participants

89 participants (51 Asian American and 38 non-AA participants) completed the pre-survey and post-survey through our various recruitment methods (audience members of the live show, community social media, students at the university, and the online platform Prolific). While we attempted to recruit participants who identified as Asian American, Pacific Islander, and Desi American, our final sample only included individuals who identified as Asian American, as there was a lack of response from Pacific Islander and Desi Americans. Most participants identified as Asian/Asian American (57.3%), with 40.4% identifying as White and 2.2% as Black or African American. The average age of participants was 27.62 (SD = 9.82). Most participants identified as female (67.4%), heterosexual (79.3%), single (51.7%), and with no diagnosis of mental health concerns (68.5%). Table 1 provides further detailed demographic information of participants by groups (AA and non-AA).

**Table 1:** Demographic information of viewers of the special edition this is my brave: stories from the Asian, Pacific islander, desi American community show

	AA^a^	non-AA^a^	Total	Group comparison
N	51	38	89	
Age (SD)	32.35 (9.71)	21.26 (5.43)	27.62 (9.82)	*p* <.001
Gender				*p* <.05
Male (%)	22 (43.1)	4 (10.5)	26 (29.2)	
Female (%)	27 (52.9)	33 (86.8)	60 (67.4)	
Non-Binary/Other (%)	2 (4.0)	1 (2.6)	3 (3.3)	
Sexual Orientation				
Heterosexual (%)	39 (76.5)	32 (84.2)	71 (79.3)	
Bisexual (%)	4 (7.8)	5 (13.2)	9 (10.1)	
Gay/Lesbian/Queer (%)	4 (7.8)	0	4 (4.5)	
Questioning (%)	1 (2.0)	0	1 (1.1)	
Asexual (%)	1 (2.0)	1 (2.6)	2 (2.2)	
Something Else (%)	2 (3.9)	0	2 (2.2)	
Education				*p* <.001
High School Degree (%)	9 (17.6)	5 (13.2)	14 (15.7)	
Some College but No Degree (%)	4 (7.8)	22 (57.9)	26 (29.2)	
Associate Degree (%)	4 (7.8)	8 (21.1)	12 (13.5)	
Bachelor’s Degree (%)	20 (39.2)	1 (2.6)	21 (23.6)	
Graduate or Professional Degree (%)	14 (27.5)	2 (5.3)	16 (18.0)	
Relationship Status				*p* <.05
Single (%)	26 (51.0)	20 (52.6)	46 (51.7)	
In a Relationship (%)	10 (19.6)	17 (44.7)	27 (30.3)	
Married or Domestic Partnership (%)	13 (25.5)	1 (2.6)	14 (15.7)	
Divorced (%)	2 (3.9)	0	2 (2.2)	
Diagnosis of Mental Illness				*p* <.001
Yes (%)	7 (13.7)	19 (50.0)	26 (29.2)	
No (%)	42 (82.4)	19 (50.0)	61 (68.5)	
Unsure (%)	2 (3.9)	0	2 (2.2)	
Past Treatment				*p* <.05
Yes (%)	11 (21.6)	18 (47.4)	29 (32.6)	
No (%)	39 (76.5)	20 (52.6)	59 (66.3	
Unsure (%)	1 (2.0)	0	1 (1.1)	
Current Treatment				*p* <.05
Yes (%)	4 (7.8)	10 (26.3)	14 (15.7)	
No (%)	46 (90.2)	28 (73.7)	74 (83.1)	
Unsure (%)	1 (2.0)	0	1 (1.1)	

^a^ Asian American

Independent samples t-tests and chi-squares were utilized to examine differences between groups (AA and non-AA). Independent samples t-tests showed a significant difference in age between the two groups. Chi-square tests showed a significant difference in gender, relationship status, education, mental health diagnosis, past treatment, and current treatment between groups. There were no differences in sexual orientation between groups.

### Effects of APIDA Storytelling from Pre- to Post-Intervention

Each measure's means and standard deviations at pre-and post-test are summarized in Table 2, along with the Cronbach’s alphas for each measure obtained from this sample. To explore the impact of the intervention on our variables of interest, a paired-sample t-test was conducted to compare pre-intervention, and post-intervention means for each dependent variable (public stigma, perceived stigma, anti-Asian stereotypes, and intentions to seek care). We found that intentions to seek care were significantly higher for post-intervention scores (M = 36.30, SD = 10.47) as compared to pre-intervention scores (M = 33.81, SD = 10.30; *t*(88) = −5.38, *p* <.001). Paired-sample *t*-tests showed a significant decrease in personal mental illness stigma (*t*(88) = 6.06, *p* <.001) from pre-intervention (M = 35.13, SD = 9.91) to post-intervention (M = 30.69, SD = 9.09), as well as a significant decrease in perceived mental illness stigma (*t*(88) = 3.03, *p* <.05) from pre-intervention (M = 51.15, SD = 10.19) to post-intervention (M = 48.25, SD = 10.97).

Anti-Asian American stereotypes were measured using a total score and two sub-scores of sociability and competence. We found a significant decrease in overall anti-Asian American stereotypes (*t*(88) = 2.45, *p* =.02) from pre-intervention (M = 59.31, SD = 18.23) to post-intervention (M = 56.52, SD = 19.84). There was also a significant decrease in scores of the sociability subscale (*t*(88) = 2.39, *p* =.02) from pre-intervention (M = 27.93, SD = 10.10) to post-intervention (M = 26.45, SD = 11.83). However, there were no differences between pre-and post-intervention for the competence subscale.

**Table 2:** Comparisons of stigma, racism, and stereotype measures from pre-to post-Intervention for all participants

Measure	Cronbach’s α of scale		Pre-Test		Post-Test		t	Effect size (d)	95% LCL	95% UCL
	Pre	Post	M	SD	M	SD				
Personal Stigma	0.72	0.68	35.13	9.91	30.69	9.09	6.06**	0.64	2.99	5.91
Perceived Stigma	0.87	0.91	51.15	10.19	48.25	10.97	3.03*	0.32	0.99	4.80
Care Seeking Intentions	0.81	0.83	33.81	10.30	36.30	10.47	−5.38**	− 0.57	−3.42	−1.57
Anti-Asian American stereotypes (total)	0.91	0.93	59.31	18.23	56.52	19.84	2.45*	0.26	0.53	5.07
SAAAS^a^ Sociability subscale	0.87	0.93	27.93	10.10	26.45	11.83	2.39*	0.25	0.25	2.72
SAAAS^a^ Competence subscale	0.88	0.86	31.38	10.88	30.07	10.34	1.86	0.20	− 0.08	2.71

Note ** *p* <.001 * *p* <.05

^a^ Scale of Anti-Asian attitudes and stereotypes

### Comparison of Means Between Asian and Non-Asian Individuals

To examine our hypothesis that the effect of the intervention would be significantly more significant for AA individuals than non-AA individuals, we conducted a within-between ANCOVA controlling for age, gender, education, relationship status, mental health diagnosis, past treatment, and current treatment. There was no significant time by AA identity interaction effect for personal stigma, perceived stigma, anti-racism, care-seeking intentions, or anti-Asian American stereotypes. However, we did find several significant main effects of time on the outcome variables after controlling for age, gender, education level, relationship status, mental health diagnosis, past treatment, and current treatment. There was a significant main effect of time on perceived stigma (F(1,80) = 4.24, *p* =.04). There was a significant effect of time on overall anti-Asian American stereotypes (F(1,80) = 7.83, *p* =.01). There was also a significant main effect of time on the sociability sub score of the Anti-Asian American Stereotypes scale (F(1, 80) = 8.41, *p* =.01) and the competence sub score (F(1, 80) = 4.02, *p* =.05). After controlling for the same covariates, we did not find any significant main effects of time for public stigma or care-seeking intentions. Table 3.

**Table 3:** Comparison between groups controlling for covariates

Measure	Mean pre/post differences [95%CI]				F^a^	Effect size (η_*p*_^2^)	
	Asian American [LL, UL] (*n* = 51)		non-Asian American [LL, UL] (*n* = 38)				
	Pre	Post	Pre	Post			
Personal Stigma	35.88 [32.82, 38.93]	32.06 [29.24, 34.87]	34.14 [30.46, 37.83]	28.84 [25.45, 32.24]	0.55	0.01	
Perceived Stigma	50.71 [47.99, 53.92]	48.02 [44.53, 51.50]	51.73 [47.86, 55.61]	48.56 [44.35, 52.77]	0.04	< 0.001	
Care Seeking Intentions	32.43 [29.27, 35.60]	35.80 [32.56, 39.03]	35.66 [31.84, 39.47]	36.99 [33.08, 40.89]	2.55	0.03	
Anti-Asian American Stereotypes	63.99 [58.30, 69.68]	63.00 [57.06, 68.93]	53.04 [46.17, 59.90]	47.82 [40.66, 54.99]	2.03	0.03	
SAAAS- Sociability	30.82 [27.69, 33.94]	30.03 [26.48, 35.58]	24.06 [20.29, 27.83]	21.67 [17.36, 25.93]	1.05	0.01	
SAAAS - Competence	33.17 [29.69, 36.66]	32.97 [29.74, 36.19]	28.98 [24.77, 33.19]	26.18 [22.29, 30.07]	1.99	0.02	

* *p* <.05, ** *p* <.001

All F-tests controlled for age, gender, relationship status, education level, mental health diagnosis, past treatment, and current treatment SAAAS = scale of anti-Asian American stereotypes

## Discussion

This study examines the impact of stories from APIDA members living with a mental illness on mental illness stigma and racial/ethnic bias towards Asian American community members. Our study provides preliminary evidence that viewing stories from APIDA individuals living with a mental illness reduced mental illness-related stigma (public stigma and perceived stigma) and improved attitudes towards treatment-seeking. This is consistent with past research on TIMB, which has shown that TIMB stories are effective in reducing stigma and improving treatment-seeking attitudes (Conner et al., 2023; Kosyluk et al., 2018, 2021). These results provide further support of the impact of TIMB as a mental illness stigma reduction program. They add to the literature preliminary evidence that narratives that are culturally adapted, such as those developed for the TIMB: SAC show, have implications for broader use in reducing mental illness stigma and may be salient for Asian American communities (Conner et al., 2023; Kosyluk et al., 2021). Future research should examine the impact of TIMB: SAC stories utilizing a national sample and a randomized control trial.

Furthermore, the preliminary outcomes of this study show promise that the TIMB: SAC has some positive impacts on addressing anti-Asian stereotypes. Historically, the Model Minority stereotype reinforces the view that individuals in the AA community are competent but lack sociability (Lin et al., 2005). This point of view portrays individuals in the AA community as hard-working but with insufficient interpersonal skills; therefore, they may need to be more approachable. These prejudices can impact how the dominant group perceives the individuals in the outgroup. Our findings show a decrease in the sociability subscale of the SAAAS. After viewing the TIMB: SAC show, participants perceived individuals in the AA community as warmer. This was a particularly salient measure to include, as during the COVID-19 pandemic, there has been a significant increase in violence and discrimination toward the APIDA community (Gao & Sai, 2020; Korn, 2021). It is essential to address experiences of discrimination among minoritized racial and ethnic groups, as these experiences have been linked to mental health outcomes in communities of color (Okazaki, 2009; Schouler-Ocak et al., 2021).

Our findings indicated that although scores for each measure moved in a positive direction, there were no significant differences between AA individuals and non-AA individuals. This was unexpected as prior work indicates that stigma change programs are most effective when participants view stories from individuals who are similar in identity (Michaels et al., 2015). While this was an unexpected finding, the overall impact of viewing TIMB: SAC regardless of racial/ethnic identity was a decrease in mental illness stigma. Some implications for these results could be a need for more education on stigma and discussion with the AA community to help decrease mental health stigma and seek help when needed. Future research should include qualitative interviews and focus groups to understand how mental illness is currently viewed within this community. Additionally, it will be necessary to hear feedback from the different subgroups of the AA community, which encompasses a wide range of ethnicities and variability in cultural beliefs and values.

### Limitations and Future Directions

Our study is not without limitations. First, most research participants were Florida state residents, so our findings may not be generalized to other geographic areas. Participant selection also came from various settings (nationally, on social media, and among university students), limiting our findings’ generalizability. While we intended to recruit APIDA participants, only Asian American participants responded. Our sample was small and therefore all Asian American participants were aggregated; however, it is important to note that these groups are heterogeneous and have different discrimination and stigma experiences that could impact mental health and mental health outcomes. Future research should look to explore the differences within the AA community and mental health. Another limitation is that there was no randomization or comparison group. Therefore, future studies should be conducted from a more nationally representative sample and draw a comparison of TIMB: SAC to a control. However, despite these limitations, this study has implications for using stories such as those from TIMB: SAC as a tool to decrease mental health stigma and anti-Asian stereotypes and beliefs.

## Conclusion

This evaluation of This Is My Brave: Stories from the APIDA community examines how culturally meaningful stories can impact mental illness stigma, intentions of seeking mental health care, anti-Asian attitudes, and anti-racism. This work has implications for the use of stories from APIDA members for mental health practitioners and community agencies addressing the stigma affecting Asian American communities.

## Data Availability

The data supporting this article’s findings cannot be publicly available due to participant private information, but are available from the corresponding author upon reasonable request.
